# A systematic review of cross-cultural adaptation of the National Institutes of Health Chronic Prostatitis Symptom Index

**DOI:** 10.1186/s12955-021-01796-8

**Published:** 2021-05-31

**Authors:** Rong-liang Dun, Jennifer Tsai, Xiao-hua Hu, Jian-min Mao, Wen-jing Zhu, Guang-chong Qi, Yu Peng

**Affiliations:** 1 Urology Surgery, Yueyang Hospital of Integrated Traditional Chinese and Western Medicine Hospital, Shanghai University of Traditional Chinese Medicine, 110 Ganhe Road, Shanghai, 200437 China; 2 Karolinska Institute, Stockholm, Sweden; 3 Urology Surgery, Shanghai Seventh People’s Hospital, Shanghai, China

**Keywords:** Cross-cultural adaptation, Measurement property, National institutes of health chronic prostatitis symptom index, Systematic review, Translation

## Abstract

**Background:**

The National Institutes of Health Chronic Prostatitis Symptom Index (NIH-CPSI) was developed to accurately assess the pain, urinary symptoms, and quality of life related to chronic prostatitis/chronic pelvic pain syndrome (CP/CPPS). This study aimed to evaluate the cross-cultural adaptations of the NIH-CPSI.

**Method:**

PubMed, Embase, CINAHL, and SciELO databases were searched from their established year to September 2020. Cross-cultural adaptations and the quality control of measurement properties of adaptations were conducted by two reviewers independently according to the *Guidelines for the Process of Cross-Cultural Adaptation of Self-Report Measures* and *the Quality Criteria for Psychometric Properties of Health Status Questionnaire.*

**Results:**

Area total of 21 papers with 16 adaptations, and six studies of the original version of the NIH-CPSI were enrolled in the systematic review. Back translation was the weakest process for the quality assessment of the cross-cultural adaptations of the NIH-CPSI. Internal consistency was analyzed for most of the adaptations, but none of them met the standard. Only 11 adaptations reported test reliability, then only the Arabic-Egyptian, Chinese-Mainland, Danish, Italian, Persian, and Turkish adaptations met the criterion. Most adaptations reported the interpretability, but only the Danish adaptation reported the agreement. The other measurement properties, including responsiveness, and floor as well as ceiling effects were not reported in any of the adaptations.

**Conclusions:**

The overall quality of the NIH-CPSI cross-cultural adaptations was not organized as expected. Only the Portuguese-Brazilian, Italian, and Spanish adaptations reached over half the process for the cross-cultural adaptation. Only the Turkish adaptations finished half of the measurement properties of cross-cultural adaptations.

**Supplementary Information:**

The online version contains supplementary material available at 10.1186/s12955-021-01796-8.

## Introduction

Chronic prostatitis/chronic pelvic pain syndrome (CP/CPPS) is a common disorder among men [1]. It is defined as chronic pelvic pain not caused by other identifiable pathologies and is often characterized by with urogenital pain, lower urinary tract symptoms, psychological issues, and sexual dysfunction [2, 3]. Men of all ages and races may experience prostatitis, with a worldwide prevalence of 2% to 10% [4]. The CP/CPPS causes morbidity, through both symptoms and associated impairment in health-related quality of life, thus illustrating the importance of patient-centered outcomes. Moreover, CP/CPPS is a poorly-defined clinical entity, and therefore is prone to misdiagnosis, mistreatment, and mismanagement [5]. The lack of a systematized and universally accepted outcome measure has led to inconsistent and vague results in CP/CPPS studies while making patient evaluation a challenge, as well as hindering research and clinical endeavors in aiding patients with CP/CPPS, thus The National Institutes of Health (NIH) Chronic Prostatitis Collaborative Research Network developed the NIH Chronic Prostatitis Symptom Index (NIH-CPSI) by Litwin and co-workers in 1999, in order to accurately assess the extent of CPPS, objectively measure the symptoms in natural history studies, and to assess the outcome parameters in clinical trials [6].

The NIH-CPSI, a self-administered questionnaire has nine items, divided into three domains: pain or discomfort (with a total score ranging from 0 to 21), urinary symptoms (with a total score ranging from 0 to 10), and impact on the quality of life (QOL) (with a total score ranging from 0 to 12 points) [6]. It is used as a diagnostic tool for the diagnosis and follow-up of CP/CPPS. In previous studies, the NIH-CPSI was shown to be reliable, valid, and responsive to change [7–10]. Pain scores of perineal or ejaculatory discomfort ≥ 8 are good predictors of moderate to severe CP/CPPS [11]. The scale was also used by English speakers with different cultural backgrounds, such as Australian, Malaysian, and Spanish, and found to have good concurrent validity, and discriminant validity [12–14].

Initially it was written in English, but in the present day, it has been translated into many other languages including Arabic, and Chinese. Due to the cultural differences, a simple translation of the original version of a questionnaire does not guarantee similar measurement properties and rough translations may lead to construct bias, method bias, and item bias, all of which impact the validity of cross-cultural comparisons [15, 16]. Whether the NIH-CPSI has similar reliability and validity as the logical cross-cultural adaptation of the original edition is still uncertain. Therefore, a systematic review on the quality of the cross-cultural adaptations of the NIH-CPSI is necessary.

## Materials and methods

### Study selection

The search for articles was performed in the PubMed, Embase, CINAHL and SciELO from their established year to September 2020. The search terms included “National Institutes of Health Chronic Prostatitis Symptom Index”, “NIH-CPSI”, “NIH Chronic Prostatitis Symptom Index”, “cross-cultural”, “equivalence”, “translation”, “validation”, and “adaptation”. Additional hand searching of journals, references lists, conference papers, and textbooks related to the NIH-CPSI were performed comprehensively. There was no language restriction.

### Inclusion and exclusion criteria

The following studies were included:

1. Studies related to the cross-cultural adaptation development of the NIH-CPSI;
2. Studies reporting the process of cross-cultural adaptations;
3. Studies on the quality assessment of at least one measurement criterion of a cross-cultural adaptation;
4. Otherwise, other validation studies from different English-speaking societies were also included.

Emails were also sent to the authors asking for their publications that were not available in full free of charge. Studies not reporting the detailed adaptation process were excluded. Two reviewers checked the potentially relevant studies according to the inclusion and exclusion criteria, and selected eligible studies independently. Any disagreement was resolved through discussion with the third reviewer.

### Data extraction and quality assessment

The language, population, publication year, and other related information about the studies were extracted by two independent reviewers in a predefined form. Then the third reviewer verified the information.

Quality assessment was made by two reviewers independently. The results were adopted on the premise that the weighted kappa (κ) was more than 0.75. Any disagreement was resolved by consensus, if a consensus could not be reached; a third reviewer decided the result.

The translation and cross-cultural adaptation methods of each study were classified according to the *Guidelines for the Process of Cross-Cultural Adaptation of Self-Report Measures* [17]. First, independent initial translations should be performed by a translator who is familiar with the field of medicine and by another translator with a non-medical background. These two independently performed translations (T1 and T2) should be synthesized (T1-2). Next, two different translators who are native English speakers and unfamiliar with the outcome measurement tool should provide a back translation into English independently (B1 and B2). Then, the next step is an expert committee made up of methodologists, health professionals, language professionals, and translators to review the original questionnaire as well as each translation (T1, T2, T12, B1, and B2). This committee then agrees on any changes that need to adapt to the tool and creates a new draft version of the questionnaire. The prefinal version should then be tested with at least 30–40 patients from the target setting. This phase is important to identify the understanding, and acceptable and emotional impact of the questionnaire items, besides detecting items that were confusing or misunderstood. Finally, the final version of the questionnaire is appraised by the expert committee again, and they should unanimously approve the final version of the tool. These procedures are described with more detail in Additional file 1: Table S1.

The measurement properties were assessed according to the *Quality Criteria for Psychometric Properties of Health Status Questionnaire*, which focused on assessing of the psychometric properties [18]. The evaluation in this study included content validity, construct validity, internal consistency, criterion validity, concurrent validity, discriminant validity, agreement, reliability, responsiveness, and ceiling and floor effects, as well as interpretability. A clear description of measurement objectives, concept to be measured, project selection, target population participation means a positive rating for content validity. A positive rating for internal consistency was assigned when factor analysis was applied and Cronbach’s α was found to be from 0.70 to 0.95, with the sample size is greater than 7 * items and a minimum number of 100 subjects. Although CP is a common complaint in societies, but no golden standard exists. Therefore, all the adaptations lacked criterion validity. Reliability refers to the extent to which patients can be distinguished from each other despite measurement error (relative measurement error). Generally, an intraclass correlation coefficient (ICC) of > 0.7 is recommended as a minimum standard for reliability [18]. These procedures are described in more detail in Additional file 2: Table S2.

## Results

**Fig. 1:**
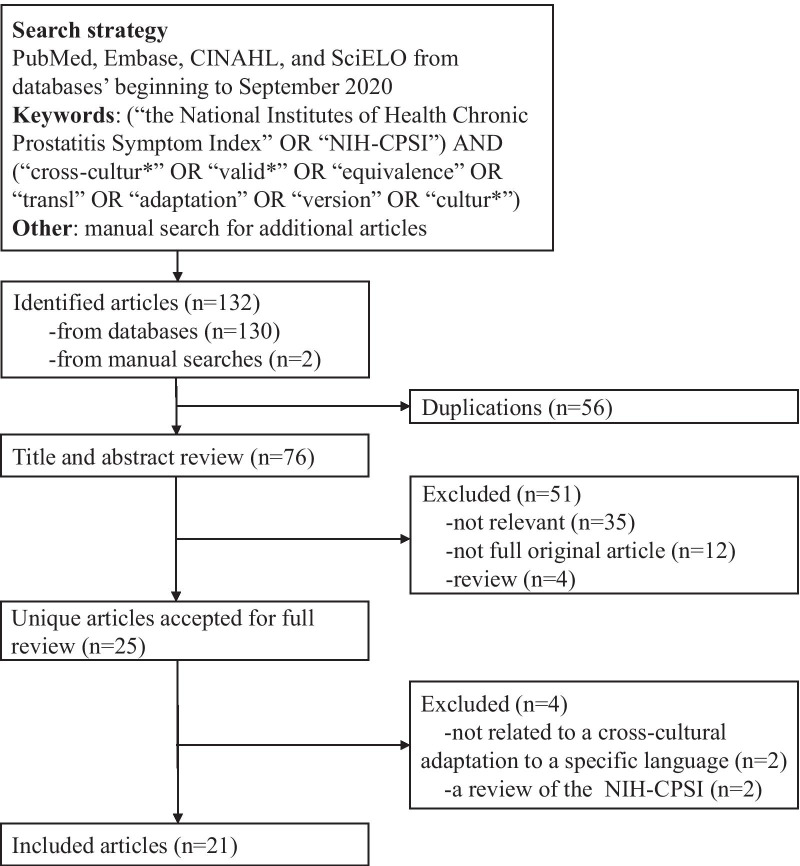
Flowchart of the search process of this review

A total of 132 studies were identified in the initial search. Of these, 56 publications were excluded because of duplications, and 35 were not relevant. Further, 12 studies were intervention trials, and 4 were reviews. The last 25 studies were identified as potentially relevant publications after screening by titles and abstracts (Fig. 1).

Two studies were excluded due to subsequent research of the original NIH-CPSI without testing measurement properties [19, 20], and two studies were reviews of the NIH-CPSI [7, 21] after full-text selection. Among the 21 studies included, 16 cross-cultural adaptations of the NIH-CPSI were in 15 different languages/cultures [13, 14, 22–35]; otherwise, four studies related to the original version in the US, and one in Australia, one in Spanish, and one in Malaysian were also included [7, 9–14]. There were two adaptations in Japanese [31, 32]. Besides, two multiple were performed studies in German (1,2) [28, 29] (Table 1).

**Table 1:** Description of cross-cultural adaptations for the National Institutes of Health Chronic Prostatitis Symptom Index

Language-population	Year	Sample size	Sample size calculation	Consecutive or not	Time interval of test–retest reliability
Original-American [7, 9–11]	1999/2003/2006	261 CPPS/151 CP, 149 BPH, 134 healthy controls/ 174 CP/CPPS/a randomly selected cohort of white men 47 to 90 years old	No/No/No/No	No/No/No/No	1 month/ 6 weeks
Original-Australian [12]	2009	Australian men aged 16–64 years	No	No	NA
Original-Malaysian [13]	2006	47 CP/CPPS, 20 BPH, 13 healthy controls	No	No	NA
Original-Spanish [14]	2001	37 CP	No	No	NA
Arabic-Egyptian [22]	2006	30 CPPS, 48 healthy controls	No	Yes	1 week
Chinese-Mainland [23]	2010	162 CP, 97 healthy controls	No	Yes	4–6 h
Chinese-Malaysian [13]	2006	32 CP/CPPS, 43 BPH, 72 healthy controls	No	No	Short-term test–retest was evaluated 1 week later, long-term 14 weeks later
Danish [24]	2019	112 CP/CPPS	No	No	Four days
Estonian [25]	2006	64 CP/CPPS, 73 controls without CP/CPPS	No	No	NA
Finnish [26]	2003	155 CPPS, 12 controls had no previous urological history	No	Yes	< 1 week later
French-Canadian [27]	2005	36 CP/CPPS, 38 controls presented for pre-vasectomy consultation	No	Yes	14 days
German (1,2) [28, 29]	2001, 2004	137 patients with CP/CPPS NIH type III	No	Yes	NA
Italian [30]	2005	42 CPPS, 81 healthy controls	No	No	1 week later
Japanese (a) [31]	2002	34 patients with CP/CPPS NIH type III, 35 BPH, 18 controls	No	No	NA
Japanese (b) [32]	2002	103 CP, 60 BPH, 87 healthy controls	No	No	2 weeks
Malaysian [13]	2006	21 CP/CPPS, 8 BPH, 12 healthy controls	No	No	Short-term test–retest was evaluated 1 week later, long-term 14 weeks later
Persian [33]	2020	42 CP/CPPS, 38 healthy controls	No	Yes	1 week
Portuguese-Brazilian [34]	2013	30 CPPS	No	Yes	1 h
Spanish [14]	2001	37 CP	No	Yes	NA
Turkish [35]	2020	116 CP/CPPS, 88 healthy controls	No	No	2 weeks

CP, chronic prostatitis; CPPS, chronic pelvic pain; BPH, benign prostatic hyperplasia; NA = not available; German (1,2), two German publications with the same adaptation; Japanese (a) and Japanese (b), two different Japanese adaptations

The sample size of the studies on validity ranged from 30 to 434, but none of them reported sample size calculation. The Original-American, Chinese-Malaysian, Japanese (a), Japanese (b) and Malaysian adaptations enrolled patients with CP/CPPS, patients with benign prostatic hyperplasia (BPH) patients, and healthy controls [9, 13, 31, 32]. The Arabic-Egyptian, Chinese-Mainland, Estonian, Finnish, French-Canadian, Italian, Persian, and Turkish adaptations enrolled patients with CP/CPPS, and healthy controls, but patients with BPH [22, 23, 25–27, 30, 33, 35]. The Original-American, Danish, German (1,2), Portuguese-Brazilian, and Spanish adaptations included only patients with CP/CPPS [7, 11, 14, 24, 28, 29, 34]. More than half of the applications included consecutive patients [14, 22, 23, 26–29, 33, 34].

### Quality assessment of the cross-cultural adaptations of the NIH-CPSI

The quality assessment of the adaptation process was assessed by two independent reviewers achieving a κ of 0.876. A consensus was achieved on 100% of occasions, when the reviewers had disagreements (Table 2).

**Table 2:** Quality assessment of the process for cross-cultural adaptations of the National Institutes of Health chronic prostatitis symptom

Language-population	Forward translation	Synthesis	Back translation	Expert committee review	Pretesting	Appraisal of the Adaptation Process
Arabic-Egyptian [18]	+	+	–	–	0	+
Chinese-Mainland [19]	–	+	?	0	0	+
Chinese-Malaysian [20]	?	+	–	0	0	+
Danish [21]	?	+	+	–	–	+
Estonian [22]	–	+	–	–	+	+
Finnish [23]	–	+	–	+	?	+
French-Canadian [24]	–	+	–	0	–	+
German (1,2) [25, 26]	?	+	–	?	0	+
Italian [27]	+	+	–	+	?	+
Japanese (a) [28]	–	+	0	0	0	+
Japanese (b) [29]	–	+	–	0	0	+
Malaysian [20]	?	+	–	0	0	+
Persian [30]	–	+	–	?	0	+
Portuguese-Brazilian [31]	+	+	+	+	+	+
Spanish [32]	–	+	–	+	+	+
Turkish [33]	?	+	+	?	–	+

+  = Positive rating; ? = doubtful design or method;—= negative rating; 0 = no information available; German (1,2), two German publications with the same adaptation; Japanese (a) and Japanese (b), two different Japanese adaptations

Most adaptations reported forward translation. Only three adaptations completely met the requirement that the forward translation process should be completed by one translator with the medical background and the other one with no medical background [22, 30, 34]. The two translators of the Chinese-Mainland, Estonian, Finnish, French-Canadian, Japanese (a), and Japanese (b) adaptations had a medical background and were aware of the concepts being examined in the questionnaire [23, 25–27, 31, 32]. In contrast, none of the translators of the Persian, and Spanish adaptations were familiar with the NIH-CPSI [14, 33]. The Chinese-Malaysian, Danish, German (1,2), Malaysian, and Turkish adaptations did not explain the specific background of the translators [13, 24, 28, 29, 35].

Most of the adaptations introduced the synthesis stage of translation, and met the requirements of the synthesis process.

In this review, only the Danish, Portuguese-Brazilian, and Turkish adaptations finished back translation completely [24, 34, 35]. Most of the adaptations had only one translator [13, 14, 22, 25–30, 32, 33]. Therefore, the Chinese-Mainland adaptation did not report whether the back translators were native English speakers [23], and the Japanese (a) adaptation provided no information about back translation [31].

Only the Finnish, Italian, Portuguese-Brazilian, and Spanish adaptations met the standards of composition for the existence of an expert committee [14, 26, 34]. The German (1,2), Persian, and Turkish adaptations did not explain the specific composition of this committee [28, 29, 33, 35]. The Arabic-Egyptian adaptation enrolled only language professionals, and the Estonian adaptation only clinicians [22, 25]; the Danish adaptation did not enroll methodologists [24]. No information was found in the other adaptations [13, 23, 27, 31, 32].

The final process of adaptation process was the pretest. Only three adaptations met the requirements [14, 25, 34]. Patients were not enough for the prefinal versions of French-Canadian, Danish, and Turkish adaptations [24, 27, 35]. The Finnish and Italian adaptations did not report the sample size of patients [26, 30]. The others lacked information about this process [13, 22, 23, 28, 29, 31–33]. All adaptations had a submission of the final version.

### Methodology used for property measurement

The κ of the two reviewers was 0.869. The methodological quality and the measurement of the studies are provided in Table 3. All studies showed a clear description of the content validity in the development of a questionnaire.

**Table 3:** The measurement properties of the National Institutes of Health Chronic Prostatitis Symptom Index adaptations relate to Quality Criteria for Psychometric Properties of Health Status Questionnaire

Language-population	Content validity	Construct validity	Internal consistency	Criterion validity	Concurrent validity	Discriminant validity	Agreement	Reliability	Responsiveness	Floor and ceiling effects	Interpretability
Original-American [7, 9–11]	+	+	+	/	+	+	0	+	+	–	+
Original-Australian [12]	+	0	0	/	0	0	0	0	0	+	+
Original-Malaysian [13]	+	0	0	/	–	+	0	0	0	0	+
Original-Spanish [14]	+	–	0	/	–	0	0	–	0	0	+
Arabic-Egyptian [22]	+	0	–	/	0	+	0	+	0	0	+
Chinese-Mainland [23]	+	0	?	/	0	+	0	+	0	0	+
Chinese-Malaysian [13]	+	0	?	/	–	+	0	–	0	0	+
Danish [24]	+	0	0	/	0	0	+	+	0	0	0
Estonian [25]	+	+	+	/	0	+	0	0	0	0	+
Finnish [26]	+	+	0	/	+	+	0	0	0	0	+
French-Canadian [27]	+	–	–	/	–	+	0	–	0	0	+
German (1,2) [28, 29]	+	+	+	/	0	+	0	0	0	0	+
Italian [30]	+	0	–	/	+	+	0	+	0	0	+
Japanese (a) [31]	+	–	0	/	–	+	0	0	0	0	+
Japanese (b) [32]	+	0	–	/	0	+	0	–	0	0	+
Malaysian [13]	+	0	–	/	–	+	0	–	0	0	+
Persian [33]	+	–	–	/	0	+	0	+	0	0	+
Portuguese-Brazilian [34]	+	0	–	/	0	0	0	–	0	0	0
Spanish [14]	+	–	–	/	–	0	0	0	0	0	0
Turkish [35]	+	+	+	/	+	+	0	+	0	0	+

+  = Positive rating; ? = doubtful design or method;—= negative rating; 0 = no information available; /, no golden standard for criterion validity; German (1,2), two German publications with the same adaptation; Japanese (a) and Japanese (b), two different Japanese adaptations

The original version in American was found with qualified content validity, construct validity, internal consistency, test–retest reliability, responsiveness, discriminant validity, and interpretability [7, 9–11]. The pain dimension of the original-American NIH-CPSI had reported ceiling effects (20.70%) [9]. The original version in Australian only checked with good interpretability [12]. Original-Malaysian reported unsatisfactory internal consistency, then Original-Spanish on the contrary, since only less than 50 patients were included, the results were not convincing.

Construct validity was conducted in 10 studies, then five of them met the met the standard [9, 25, 26, 28, 29, 35], then Original-American, Estonian, Finnish, German (1,2) versions used Pearson’s *r* correlation [9, 25, 26, 29], and Turkish versions applied Spearman’s *r* correlation [35]. A negative rating was given by the reviewer for the construct validity of Original-Spanish, French-Canadian, Japanese (a), Persian, and Spanish adaptations, because their sample sizes were smaller than 100 [14, 27, 31, 33].

An analysis of the internal consistency was conducted on most of the adaptations, but only four of them met the standard [9, 25, 28, 29, 35]. A negative rating was given by the reviewer for the internal consistency of Arabic-Egyptian, French-Canadian, Italian, Japanese (b), Portuguese-Brazilian, Malaysian, Persian, and Spanish adaptations, because their sample sizes were smaller than 100 [13, 14, 22, 27, 30, 32–34]. The Chinese-Mainland, and Chinese-Malaysian adaptations did not fully meet the criteria of internal consistency for the missing factor analysis [13, 23, 28]. No information was available on the internal consistency of the Finnish and Danish adaptations [24, 26]. Only half of the adaptations reported had Cronbach’s α of more than 0.70 [14, 22, 25, 27, 32–35]. The details are shown in Table 4.

**Table 4:** The summary of the measurement properties of cross-cultural the National Institutes of Health Chronic Prostatitis Symptom Index adaptations

Language-population	Internal consistency	Construct validity	Concurrent validity	Agreement	Reliability	Responsiveness	Floor and ceiling effects	Discriminant validity				
Original-American [7, 9–11]	Total 0.82; Pain 0.65; Void 0.77; QOL 0.73	**Pain location** 0.35–0.88 **Pain frequency** 0.18–0.87 **Pain severity** 0.39–0.95 **Void** 0.32–0.73 **Impact** 0.32–0.94 **Overall QOL** 0.35–0.94	**AUA symptom index:** Pain 0.39; Void 0.87; QOL 0.46 **GCPS Pain:** Pain 0.61; Void 0.31; QOL 0.54; Total 0.60 **GCPS Pain:** Pain 0.38; Void 0.13; QOL 0.45; Total 0.40	0	Pain 0.83–0.93; Void 0.90; QOL 0.87–0.91	ROC curve 0.83	% Ceiling Effects (best possible score) 20.70 Pain	**CP/CPPS versus Healthy Individuals:** 0.67				
Original-Australian [12]	0	0	0	0	0	0	0	0				
Original-Malaysian [13]	Total 0.57; Pain 0.34; Void 0.32; QOL 0.33	0	**IPSS** Total 0.63; Pain 0.23; Void 0.85; QOL 0.44		Total 0.89; Pain 0.89; Void 0.84; QOL 0.85	0	0	**CP/CPPS versus Healthy Individuals:** Total 1.00; Pain 1.00; Void 0.87; QOL 1.00 **CP/CPPS versus BPH:** Total 0.; Pain 0.99; Void 0.39; QOL 0.75				
Original-Spanish [14]	Total 0.86; Pain 0.86; Void 0.79; QOL 0.87	Total 0.89–0.97; Pain 0.80–0.97; Void 0.76–0.89; QOL 0.76–0.94	0	0	0	0	0	0				
Arabic-Egyptian [22]	Total 0.92; Pain 0.89; Void 0.92; QOL 0.87	0	0	0	Total 0.90; Pain 0.84; Void 0.92; QOL 0.94	0	0			CPPS	Controls	*P*
									Total	22.3(4.1)	2.3(2.8)	< 0.001
									Pain	9.3(2.5)	0.6(0.2)	< 0.001
									Void	3.7(1.8)	0.7(0.4)	< 0.001
									QOL	7.3(2.4)	0.7(0.2)	< 0.001
Chinese-Mainland [23]	Total 0.61; Pain 0.71; Void 0.59; QOL 0.75	0	0	0	Total 0.98; Pain 0.98; Void 0.98; QOL 0.97	0	0			CPPS	Controls	*P*
									Total	23.33(5.91)	1.95(1.97)	0.00
									Pain	8.80(4.26)	0.37(1.03)	0.00
									Void	5.30(2.82)	0.15(0.58)	0.00
									QOL	9.23(1.90)	1.42(1.20)	0.00
Chinese-Malaysian [13]	Total 0.63; Pain 0.33; Void 0.73; QOL 0.60	0	**IPSS** Total 0.72; Pain 0.35; Void 0.78; QOL 0.29	0	Total 0.9; Pain 0.80; Void 0.82; QOL 0.77	0	0	**CP/CPPS versus Healthy Individuals:** Total 1.00; Pain 1.00; Void 0.90; QOL 0.99 **CP/CPPS versus BPH:** Total 0.87; Pain 0.96; Void 0.47; QOL 0.68				
Danish [24]	0	0	0	No signs of systematic bias in Bland Altman Plot	Total 0.93 (0.91–0.96); Pain 0.88 (0.84–0.92); Void 0.91 (0.88–0.94); QOL 0.93 (0.91–0.96)	0	0	0				
Estonian [25]	Total 0.82; Pain 0.67; Void 0.63; QOL 0.78	Total 0.62–0.93; Pain 0.35–0.92; Void 0.35–0.62; QOL 0.47–0.93	0	0	0	0	0			CPPS	Controls	*P*
									Total	15.3 (5.6)	6.0 (6.7)	< 0.001
									Pain	8.6 (2.5)	2.2 (3.3)	< 0.001
									Void	1.2(1.4)	1.3 (1.9)	0.86
									QOL	6.1 (2.1)	2.5(3.0)	0.001
Finnish [26]	0	Pain 0.36–0.89; Void 0.36–0.67; QOL 041–0.71	**VAS** Total 0.76; Pain 0.87; Void 0.27; QOL 0.60	0	0	0	0			CPPS	Controls	*P*
									Total	20.2 (8.6)	5.8 (4.5)	< 0.001
									Pain	9.9 (4.5)	0.1 (0.3)	< 0.001
									Void	3.9 (3.1)	0.3 (0.6)	< 0.001
									QOL	6.5 (2.9)	5.5 (4.3)	> 0.05
French-Canadian [27]	Pain 0.807; Void 0.570; QOL 0.884	Pain 0.67–0.94; Void 0.55–0.67; QOL 0.62–0.0.97	**SF− 12 Mental scale** Pain − 0.29 to − 0.44; Void − 0.28 to − 0.43; QOL − 0.41 to − 0.51 **SF− 12 Mental scale** Pain − 0.32 to − 0.50; Void − 0.20 to − 0.37; QOL − 0.40 to − 0.59	0	0.352–0.862	0	0			CPPS	Controls	*P*
									Pain	8.48 (4.67)	1.72 (2.27)	< 0.001
									Void	3.33 (2.31)	0.95 (1.12)	< 0.001
									QOL	5.39 (2.84)	0.76 (1.81)	> 0.05
German (1,2) [28, 29]	Total 0.74; Pain 0.60; Void 0.69; QOL 0.67	Total 0.63–0.85 Pain 0.24–0.85; Void 0.24–0.63; QOL 0.32–0.77	0	0	0	0	0			NIH IIIA	NIH IIIB	*P*
									Total	20(21.4)	24(23.9)	0.03
									Pain	11(10.1)	12(11.3)	0.09
									Void	3(3.2)	3(3.8)	0.14
									QOL	8(8)	9(8.7)	0.16
Italian [30]	Total 0.95; Pain 0.84; Void 0.96; QOL 0.86	0	**VAS** Total 0.80; Pain 0.88; Void 0.22; QOL 0.71 **IPSS** Total 0.55; Pain 0.27; Void 0.94; QOL 0.25	0	Total 0.90; Pain 0.83; Void 0.82; QOL 0.93	0	0			CPPS	Controls	*P*
									Total	21.8 (8.5)	2.0 (2.8)	< 0.001
									Pain	10.5 (4.5)	0.5 (1.2)	< 0.001
									Void	4.5 (2.9)	0.9 (1.2)	< 0.001
									QOL	6.8 (3.1)	0.6 (1.0)	< 0.001
Japanese (a) [31]	0	Pain 0.662; Void 0.531	0	0	0	0	0		CP/CPPS	BPH	Controls	*P*
								Pain	9.79 (3.10)	1.66 (3.05)	0.39 (0.92)	0.00
								Void	3.82 (3.12)	3.29 (2.08)	0.72 (0.83)	0.00
								QOL	8.21 (2.23)	4.17 (1.86)	1.39 (1.38)	0.00
Japanese (b) [32]	Pain 0.83; Void 0.97; QOL 0.87	0	0	0	Pain 0.63; Urinary symptoms 0.91; QOL 0.72	0	0		CP	BPH	Controls	*P*
								Pain	9.0 (0–18)	1.0 (− 16)	0.0 (0–12)	0.00
								Void	3.0 (0–10)	4.0 (0–10)	0.0 (0–8)	0.00
								QOL	7.0 (2–11)	5.0 (0–12)	1.0 (0–6)	0.00
Malaysian [13]	Total 0.62; Pain 0.43; Void 0.83; QOL 0.45	0	**IPSS** Total 0.49; Pain − 0.18; Void 0.87; QOL 0.16	0	Total 0.80; Pain 0.94; Urinary symptoms 0.87; QOL 0.85	0	0	**CP/CPPS versus Healthy Individuals:** Total 1.00; Pain 1.00; Void 0.83; QOL 0.98				
								**CP/CPPS versus BPH:** Total 0.94; Pain 0.96; Void 0.58; QOL 0.78				
Persian [33]	Total 0.865; Pain 0.853; Void 0.652; QOL 0.726	Total 0.666–0.889; Pain 0.389–0.889; Void 0.389–0.666; QOL 0.433–0.846	0	0	Total 0.901; Pain 0.894; Void 0.912; QOL 0.846	0	0			CP/CPPS	Controls	*P*
									Total	22.47(6.9)	2.1(2.7)	< 0.001
									Pain	10.35(3.7)	0.3(0.2)	< 0.001
									Void	4.73(1.98)	0.5(0.4)	< 0.001
									QOL	7.38(2.7)	0.8(0.4)	< 0.001
Portuguese-Brazilian [34]	Pain 0.90; Void 0.85; QOL 0.93	0	0	0	Pain 0.89–0.94; Void 0.99; QOL 0.96–0.97	0	0	/				
Spanish [14]	Total 0.94; Pain 0.87; Void 0.81; QOL 0.86	Total 0.89–0.97; Pain 0.80–0.97; Void 0.76–0.89; QOL 0.76–0.94	0	0	0	0	0	/				
Turkish [35]	Total 0.864; Pain 0.862; Void 0.819; QOL 0.762	Total 0.66–0.77; Pain 0.25–0.72; Void 0.33–0.72; QOL 0.25–0.66	**VAS** Total 0.6; Pain 0.66; Void 0.22; QOL 0.29	0	Total 0.909; Pain 0.886; Void 0.925; QOL 0.874	**IPSS** Total 0.48; Pain 0.24; Void 0.54; QOL 0.4	0	0		CP/CPPS	Controls	*P*
									Total	22.8 (7.7)	2.5 (1.7)	< 0.001
									Pain	9.5 (4.8)	1.4 (1.2)	< 0.001
									Void	6 (3.2)	0.6 (0.8)	< 0.001
									QOL	7.3 (2.3)	0.5 (0.6)	< 0.001

AUA, American Urological Association; GCPS, Graded Chronic Pain Scale; QOL, quality of life; ROC, Receiver Operating Characteristic; IPSS, International Prostate Symptom Scores; CP, chronic prostatitis; CPPS, chronic pelvic pain; BPH, benign prostatic hyperplasia; VAS, visual pain scale; SF-12, 12-item short-form Health Survey

The Finnish, Italian, and Turkish adaptation showed a good correlation with the visual analogue scale or International Prostate Symptom Score, then American Urological Association symptom index a good correlation with Original-American [9, 26, 30, 35]. Therefore, a positive rating for concurrent validity was given to the adaptation enrolling at least 50 patients, while the Chinese-Malaysian, French-Canadian, Japanese (a), Malaysian, and Spanish adaptations did not have 50 patients [13, 14, 27, 31]. The others did not have concurrent validity.

Only two publication reported the discriminant validity following the guidelines [13]. The discriminant validity between the CP/CPPS group and each of the control groups was assessed by calculating the area under the receiver operating characteristic curve (AUC). The Original-Malaysian, Chinese-Malaysian, and Malaysian NIH-CPSI reported that the AUC of CP/CPPS versus healthy individuals was more then 0.80. The AUC of Original-American NIH-CPSI was 0.67, then Original-Malaysian, and Malaysian reported good discriminant validity of more than 0.75 CP/CPPS versus BPH, except void [11, 13]. Other studies reported only the *P* value of difference between CPPS, and controls or BPH [13, 22, 23, 25–32, 35].

Only nine adaptations reported test reliability, but only the Original-American, Arabic-Egyptian, Chinese-Mainland, Danish, Italian, Persian, and Turkish adaptations met the criterion [22–24, 30, 33, 35]. The sample size for the reliability should be at least 50 patients, while the Chinese-Malaysian, French-Canadian, Japanese (b), Malaysian, and Portuguese-Brazilian adaptations enrolled less than 50 patients [13, 27, 32, 34].

Most adaptations reported the interpretability, except for Danish, Portuguese-Brazilian and Spanish adaptations [14, 24, 34]. Then, only the Danish adaptation reported the agreement [24].

Other measurements such as responsiveness, and floor and ceiling effects were not reported in any of the adaptations.

## Discussion

### Summary of evidences

The objective of this study was to assess the cross-cultural adaptation procedures and the measurement properties in each adaptation of the NIH-CPSI. Back translation was the weakest process for the quality assessment of the cross-cultural adaptations of the NIH-CPSI. The main reason was the presence of only one translator in most of the adaptations. An analysis of the internal consistency was conducted on most of the adaptations, but none of them met the standard. Only 11 adaptations reported test reliability, but only the Arabic-Egyptian, Chinese-Mainland, Danish, Italian, Persian, and Turkish adaptations met the criterion. Most adaptations reported the interpretability, but only the Danish adaptation reported the agreement. The quality of several other measurement properties, including responsiveness and internal consistency was blank.

The overall quality of the NIH-CPSI cross-cultural adaptations was unsatisfactory. Only the Italian, Portuguese-Brazilian, and Spanish adaptations provided a better quality compared with the other adaptations for the quality assessment of the cross-cultural adaptations [14, 30, 34]. Only the Turkish adaptations finished half of the measurement properties [35]. Many standards were developed to measure the cross-cultural reliability of questionnaires, such as the guidelines for the process of cross-cultural adaptation of self-reported measures in 2000 [17], Consensus-based Standards for the selection of health status Measurement Instruments (COSMIN)-checklist in 2016 [36, 37], the Patient-Reported Outcomes in 2005 [38], and the Scientific Advisory Committee of the Medical Outcome Trust checklist in 1996 [39]. However, the Danish adaptation in 2019, and the Persian adaptation in 2020 showed very little improvement in the methodological quality of the cross-cultural adaptation of the NIH-CPSI [24, 33, 35].

### Sample size for the future cross-cultural adaptations of the NIH-CPSI

Many adaptations did not take pretesting of the prefinal version or did not have enough patients, which was important for adaptations. Ideally, 30 to 40 participants should be included in pretesting [17]. The patients with CP/CPPS were different from the translators, and the expert committee. Some of them did not have a high educational background, and thus the pretesting was necessary.

The sample size for the assessment of the measurement properties was also important. Additionally, 9 out of 14 adaptations reported that the internal consistency did not meet the requirement of an adequate sample size of more than 100; 5 out of 8 adaptations reported construct validity, and 5 out of 11 adaptations reported the reliability. The sample size of the studies on validity ranged from 30 to 259, but none of them reported sample size calculation. It was the most outstanding drawback for the measurement properties. Overall, 100 patients should be included in internal consistency and validity, and then 50 patients included in the reliability, agreement, and responsiveness [18]. Thus, 30 to 40 participants should be included in the pretesting process, and a sample size of at least 100 patients should be included to assess the measurement properties for NIH-CPSI.

### Best practice for evaluating the construct validity of the NIH-CPSI

The construct validity of the NIH-CPSI has been tested in most of the publications, but the method is not unified. This is best estimated using the multi-trait multi-method matrix [40]. In some cases, researchers have used either latent variable modeling or Pearson product-moment correlation based on Fisher’s Z transformation [41, 42]. An internally consistent scale is achieved through principal component analysis or exploratory factor analysis, followed by confirmatory factor analysis. A clear hypothesis exists that the factor structure is determined as pain or discomfort, urinary symptoms, and QOL, and hence confirmatory factor analysis (CFA) should be used [43, 44]. Robust maximum likelihood was used to estimate the CFA model. The fit of the model was assessed by combining the following fit indices: comparative fit index (CFI), Tucker-Lewis index (TLI), standardized root mean square residual (SRMR), and root mean square error of approximation (RMSEA). Pre-determined cut-off values were used to assess the fit (CFI and TLI > 0.95 for good fit and > 0.90 for acceptable fit; SRMR < 0.08 for good fit and < 0.12 for acceptable fit and RMSEA < 0.06 for good fit and < 0.10 for acceptable fit) [45]. It could be invalidated by too low or weak correlations with other tests, which were intended to measure the same construct. The critical values for Pearson’s or Spearman’s *r* correlations were as follows: high, *r* > 0.50; moderate, 0.50 > *r* > 0.30; and low, 0.30 > *r* > 0.25 [46]. The critical value for significant factor loading was > 0.40 [46]. According to the guidelines, the sample size for CFA should be more than 100, and 7 times of the items [47]. The CFA was conducted using the Analysis of Moment Structures Program, or the Lavaan package in R statistical software. Then, Pearson’s *r* correlations were performed using SPSS, SAS or R statistical software.

### Limitations of this review

The major English databases were included in literature retrieval. Meanwhile, manual retrieval was also shown in the references. Nonetheless, it could hardly guarantee that all cross-cultural adaptations of NIH-CPSI has been found. It was significant for a systematic review to assess all original studies that reported cross-cultural translations of the NIH-CPSI. Then, the systematic review design was defined before conducting the study as a priori, but this predefined systematic review protocol was not registered before.

## Conclusions

The overall quality of the NIH-CPSI cross-cultural adaptations was not organized as expected. Only the Portuguese-Brazilian, Italian, and Spanish adaptations reached over half of the process for the cross-cultural adaptation. Also, only the Italian and Turkish adaptations finished half of the measurement properties of cross-cultural adaptations. Future studies should consider the sample size reasonably and test responsiveness and floor and ceiling effects. Moreover, other psychometric properties should follow the guidelines.

### What is new?

1. The overall quality of the NIH-CPSI cross-cultural adaptations is not organized as expected.
2. Only the Portuguese-Brazilian and Spanish adaptations showed a better quality than the other adaptations for the quality assessment.
3. For the measurement properties, only the Italian, and Turkish adaptations finished half of the measurement properties.
4. Many standards had been developed to measure the cross-cultural reliability of questionnaires, however, from the Danish adaptation in 2019, and Persian adaptation in 2020, we could find that there was very little improvement in the cross-cultural adaptation of NIH-CPSI.

## Supplementary Information

**Additional file 1: Table S1.** Guidelines for the Process of Cross-Cultural Adaptation of Self-Report Measures

**Additional file 2: Table S2.** Quality Criteria for Psychometric Properties of Health Status Questionnaire

## Data Availability

The datasets used and/or analysed during the current study are available from the corresponding author on reasonable request.
