# Supplementary material for: A systematic review of cross-cultural adaptation of the National Institutes of Health Chronic Prostatitis Symptom Index
Source: Health Qual Life Outcomes. 2021 May 31;19:159. doi: 10.1186/s12955-021-01796-8 (PMC8166010; doi:10.1186/s12955-021-01796-8)
Supplement: Supplementary file 1 — Additional file 1: Table S1. Guidelines for the Process of Cross-Cultural Adaptation of Self-Report Measures [file 12955_2021_1796_MOESM1_ESM.doc]

Table S1. Guidelines for the Process of Cross-Cultural Adaptation of Self-Report Measures

| **Steps** | **Description** | **Rating** | **Description** |
| --- | --- | --- | --- |
| Initial Translation | At least a informed and uninformed translator independently translate to the target language | + | At least a informed and uninformed translator independently translate to the target language |
|  |  | ? | Doubtful design or method |
|  |  | - | Translation performed by only one translator |
|  |  | 0 | No information found on responsiveness |
| Synthesis of The Translations | Two forward translations to be synthesize to produce a consensus | + | Two forward translations to be synthesize to produce a consensus |
|  |  | ? | Doubtful design or method |
|  |  | - | Translation performed by only one translator or no synthesis of the translations |
|  |  | 0 | No information found on responsiveness |
| Back Translation | At least two translator with English-first language as their mother tongue that are naïve to measurement make back-translations | + | At least two translator with English-first language as their mother tongue that are naïve to measurement make back-translations |
|  |  | ? | Doubtful design or method |
|  |  | - | No back translation, or only one |
|  |  | 0 | No information found on responsiveness |
| Expert Committee | The minimum composition comprises methodologists, health professionals, language professionals, and the translators | + | The minimum composition comprises methodologists, health professionals, language professionals, and the translators |
|  |  | ? | Doubtful design or method |
|  |  | - | an expert committee not meet the need or no expert committee |
|  |  | 0 | No information found on responsiveness |
| Test of the Pre-final Version | 30 to 40 patients complete the pre-final version to probe to get at understanding of item | + | 30 to 40 patients complete the pre-final version to probe to get at understanding of item |
|  |  | ? | Doubtful design or method |
|  |  | - | Not enough person or no test of the pre-final version |
|  |  | 0 | No information found on responsiveness |
| Appraisal of the Adaptation Process | A submission of final version keep track of the translated version | + | A submission of final version keep track of the translated version |
|  |  | ? | Doubtful design or methodNo information found on responsiveness |
|  |  | 0 | No information found on responsiveness |
|  |  |  |  |

+=Positive rating; ?=doubtful design or method; -=negative rating; 0=no information available
