# Supplementary material for: A systematic review of cross-cultural adaptation of the National Institutes of Health Chronic Prostatitis Symptom Index
Source: Health Qual Life Outcomes. 2021 May 31;19:159. doi: 10.1186/s12955-021-01796-8 (PMC8166010; doi:10.1186/s12955-021-01796-8)
Supplement: Supplementary file 2 — Additional file 2: Table S2. Quality Criteria for Psychometric Properties of Health Status Questionnaire [file 12955_2021_1796_MOESM2_ESM.doc]

**Table S2.** Quality Criteria for Psychometric Properties of Health Status Questionnaire

| **Property** | **Rating** | **Quality criteria** |
| --- | --- | --- |
| Content validity | + | A clear description is provided of the measurement aim, the target population, the concepts that are being measured, and the item selection AND target population and (investigators OR experts) were involved in item selection |
|  | ? | Doubtful design or method; |
|  | - | No target population involvement |
|  | 0 | No information |
| Construct validity | + | Factor analyses performed on adequate sample size (7 * # items and ≥100), CFA CFI and TLI >0.90, SRMR <0.12, and RMSEA <0.10 OR Pearson’s r correlations > 0.3, OR factor loading > 0.4 |
|  | ? | Doubtful design or method |
|  | - | CFA CFI and TLI < 0.90, SRMR > 0.12, and RMSEA > 0.10 OR Pearson’s or Spearman’s *r* correlations < 0.3, OR factor loading < 0.4, despite adequate design and method |
|  | 0 | No information |
| Internal consistency | + | Cronbach’s alpha(s) calculated per dimension AND Cronbach’s alpha(s) between 0.70 and 0.95 |
|  | ? | Doubtful design or method |
|  | - | Cronbach’s alpha(s) ＜0.70 or ＞0.95, despite adequate design and method |
|  | 0 | No information |
| Criterion validity | + | Convincing arguments that gold standard is “gold” AND correlation with gold standard ≥0.70 |
|  | ? | Doubtful design or method |
|  | - | Correlation with gold standard ＜0.70, despite adequate design and method |
|  | 0 | No information |
| Concurrent validity | + | Specific hypotheses were formulated AND at least 75% of the results are in accordance with these hypotheses |
|  | ? | Doubtful design or method |
|  | - | Less than 75% of hypotheses were confirmed, despite adequate design and methods |
|  | 0 | No information |
| Discriminant validity | + | AUC≥0.70 OR significant differences between the two discriminant groups |
|  | ? | Doubtful design or method |
|  | - | AUC＜0.70, OR no significant differences between the two discriminant groups, despite adequate design and methods |
|  | 0 | No information |
| Agreement | + | MIC＜SDC OR MIC outside the LOA OR convincing arguments that agreement is acceptable |
|  | ? | Doubtful design or method; |
|  | - | MIC≥SDC OR MIC equals or inside LOA, despite adequate design and method; 0No information found on agreement |
|  | 0 | No information |
| Reliability | + | ICC or weighted Kappa ≥0.70 |
|  | ? | Doubtful design or method |
|  | - | ICC or weighted Kappa＜0.70, despite adequate design and method |
|  | 0 | No information |
| Responsiveness | + | SDC or SDC ＜MIC OR MIC outside the LOA OR RR＞1.96 OR AUC≥0.70 |
|  | ? | Doubtful design or method |
|  | - | SDC or SDC≥MIC OR MIC equals or inside LOA OR RR≤1.96 OR AUC＜0.70, despite adequate design and methods |
|  | 0 | No information |
| Floor or ceiling effects | + | ≤15% of the respondents achieved the highest or lowest possible scores |
|  | ? | Doubtful design or method |
|  | - | ＞15% of the respondents achieved the highest or lowest possible scores, despite adequate design and methods |
|  | 0 | No information |
| Interpretability | + | Mean and SD scores presented of at least four relevant subgroups of patients and MIC defined |
|  | ? | Doubtful design or method; |
|  | 0 | No information |
|  |  |  |

CFA, confirmatory factor analysis; CFI, comparative fit index; TLI, Tucker-Lewis index; SRMR, standardized root mean square residual; RMSEA, root mean square error of approximation; AUC, area under the receiver operating characteristic curve; MIC, minimal important change; SDC, smallest detectable change; LOA, limits of agreement; ICC, intraclass correlation; SD, standard deviation.
